# Exploring why medical students still feel underprepared for clinical practice: a qualitative analysis of an authentic on‐call simulation

**DOI:** 10.1186/s12909-021-02605-y

**Published:** 2021-03-17

**Authors:** Nichola Hawkins, Helen-Cara Younan, Molly Fyfe, Ravi Parekh, Andrew McKeown

**Affiliations:** 1 Department of Primary Care & Public Health, School of Public Health, Imperial College London, W6 8RP London, UK; 2 University of Buckingham Medical School, Hunter Street, MK18 1EG Buckingham, UK

**Keywords:** Undergraduate medical education, Preparation for practice, On‐call simulation, Cognitive load theory

## Abstract

**Background:**

Current research shows that many UK medical graduates continue to feel underprepared to work as a junior doctor. Most research in this field has focused on new graduates and employed the use of retrospective self-rating questionnaires. There remains a lack of detailed understanding of the challenges encountered in preparing for clinical practice, specifically those faced by medical students, where relevant educational interventions could have a significant impact. Through use of a novel on-call simulation, we set out to determine factors affecting perceived preparation for practice in final year medical students and identify ways in which we may better support them throughout their undergraduate training.

**Methods:**

30 final year medical students from Imperial College London participated in a 90-minute simulation on hospital wards, developed to recreate a realistic on-call experience of a newly qualified doctor. Students partook in pairs, each observed by a qualified doctor taking field notes on their decisions and actions. A 60-minute semi-structured debrief between observer and student pair was audio-recorded for analysis. Field notes and students’ clinical documentation were used to explore any challenges encountered. Debrief transcripts were thematically analysed through a general inductive approach. Cognitive Load Theory (CLT) was used as a lens through which to finalise the evolving themes.

**Results:**

Six key themes emerged from the on-call simulation debriefs: information overload, the reality gap, making use of existing knowledge, negative feelings and emotions, unfamiliar surroundings, and learning ‘on the job’.

**Conclusions:**

The combination of high fidelity on-call simulation, close observation and personalised debrief offers a novel insight into the difficulties faced by undergraduates in their preparation for work as a junior doctor. In using CLT to conceptualise the data, we can begin to understand how cognitive load may be optimised within this context and, in doing so, we highlight ways in which undergraduate curricula may be adapted to better support students in their preparation for clinical practice. Recommendations are centred around enhancing the expertise of the learner through ‘whole task’ training approaches and integrated learning, as well as navigating negative emotions and supporting lifelong ‘learning while working’.

## Background

UK medical schools are responsible for the design and delivery of training programmes that enable newly qualified doctors to practice efficiently and safely, satisfying the overarching outcome for graduates set out by the General Medical Council (GMC) [1]:

“Medical students are tomorrow’s doctors. In accordance with good medical practice, newly qualified doctors must make the care of patients their first concern, applying their knowledge and skills in a competent, ethical and professional manner and taking responsibility for their own actions in complex and uncertain situations.” (p. 7).

**Fig. 1:**
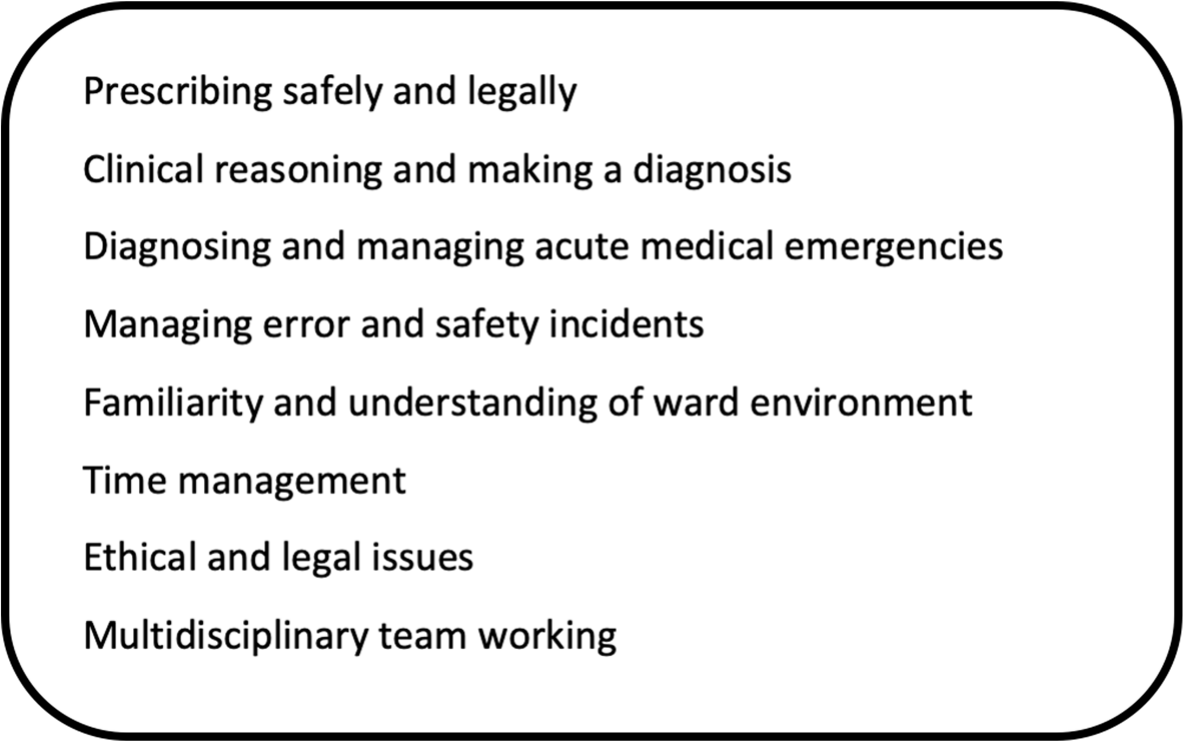
Areas of under-preparedness previously identified. UK medical graduates repeatedly report being underprepared in a number of areas of clinical practice when they start working as a junior doctor [2]

Over the last 20 years, however, we have seen a rise in research and publications that consistently describe new UK medical graduates as being inadequately prepared for clinical practice [2–5]. Despite clear documentation within the literature of specific practice areas for which graduates are repeatedly reported as being underprepared (Fig. 1), there remains a lack of detailed understanding as to why this is, and therefore how we can better support our undergraduate students in preparing accordingly.

Most research in this field has focused on new graduates and employed the use of retrospective self-rating questionnaires [2]. This methodology limits comprehensive investigation of the challenges identified, does not account for responder bias and, importantly, neglects the perspective of medical students for whom relevant educational interventions could have a significant impact.

In considering the role of observational methods in further exploring this subject area, aligned to recommendations of Monrouxe et al. [4], we subsequently describe a novel approach; a simulated on-call within the hospital setting for final year medical students with close observation and qualitative reflective debrief.

Newly qualified doctors are often apprehensive and anxious about their first on-call shifts in particular; these shifts carry with them levels of responsibility that new graduates may not have experienced before, drawing on a large number of the problematic practice areas previously described (Fig. 1). In addressing this anxiety, and facilitating the development of the relevant skills, a number of simulated on-call sessions have been developed in the UK and abroad [6–9]. Of these, the more authentic versions incorporate multiple different patient cases, across a variety of settings, with students carrying bleeps to which they must respond when called. Students consistently rank these learning experiences highly, reporting them to be valuable in their preparation for clinical practice [7, 8].

On-call simulations described in the literature rarely combine close and consistent observation of students, spanning numerous cases in different locations, with personalised reflective debrief. Furthermore, debrief following simulation has not previously been used as a qualitative method to explore the factors affecting preparedness for practice in final year medical students; most studies describe quantitative data collection via questionnaires, asking students to rate their confidence in practicing as a junior doctor before and after a simulation exercise.

We therefore present the aim of our study; to investigate why medical students continue to report under-preparedness for clinical practice, using an authentic on-call simulation. We set out to determine both the individual and environmental factors affecting preparation for practice in final year students at Imperial College School of Medicine (ICSM) and, in doing so, understand how we may better support them throughout undergraduate training for their future work as newly qualified junior doctors.

## Methods

### Simulation design and data collection

In February 2019, recent graduates from ICSM were invited to complete an online scoping survey to inform future teaching and learning within the final year of the undergraduate medical degree. The results from this survey were used to inform the design of a simulation, which was developed to recreate a realistic on-call experience for a Foundation Year 1 (FY1) doctor (i.e. a doctor in their first year of postgraduate medical training).

In September 2019, following ethical approval, the on-call simulation was used as part of a mandatory teaching session for 30 medical students, all enrolled on a year-long pilot Longitudinal Integrated Clerkship (LIC) (replacing the traditional final year of undergraduate training at ICSM). By holding the simulation at the start of the academic year, faculty sought to capture the learning needs of students at ‘baseline’ and utilise the experience to better inform subsequent final year teaching and learning activities. All 30 students consented to participation in the research study and the inclusion of their simulation debrief within the analysis. Further details of the teaching session are subsequently described.

Located within a district general hospital, the simulation commenced with a typical evening handover to the students, as they started a 90-minute out of hours shift as the FY1 doctor on-call. They carried bleeps, responding to calls from a virtual switchboard, and were required to manage multiple cases across a variety of hospital wards and clinical specialities. The simulation concluded with a night handover. Students worked in pairs and were followed throughout by an ‘observer’ – a qualified doctor taking field notes on students’ decisions and actions.

On completion of the simulation, each pair participated in a semi-structured debrief facilitated by their observer that lasted up to 60 minutes. This was recorded by dictaphone. Field notes and students’ clinical documentation were used in the debrief to explore any challenges encountered, with a focus on the skills applicable, and students’ feelings towards working as a newly qualified doctor.

### Data analysis

Audio recordings of the simulation debriefs were anonymised and transcribed. The transcripts were checked for accuracy and initially reviewed by three members of the research team (NH, HCY and AM). Following this process of data familiarisation, a selection of transcripts were independently analysed by NH and HCY, through a general inductive approach [10]. This was oriented towards identifying challenges experienced by students’ during the simulation. Code descriptions were discussed and reconciled, with any discrepancies resolved, before being applied to the entire data set.

As themes subsequently emerged, the research team discussed Cognitive Load Theory (CLT) [11] as a theoretical lens with which to conceptualise the coded data and relate findings to the wider context of medical education. CLT distinguishes between three additive sources of cognitive load associated with any given task that contribute to working memory capacity; intrinsic (essential to the task), extraneous (not essential to the task) and germane (associated with the storage of knowledge itself). Should the total cognitive load exceed the working memory capacity of an individual, there is thought to be a negative effect on both learning and performance [12]. CLT is of particular relevance to medical education, given the nature of the professional activities to be grasped by students; tasks often demand simultaneous integration of multiple and varied sets of knowledge, skills and behaviour, meaning there is a high risk of cognitive overload [12]. Themes were therefore finalised through extensive discussion between all authors, shaped and informed by CLT.

Throughout this process, we sought to maintain consideration of our differing positions, experience and research interests, and how these may have influenced our interpretation of the data. As co-leads for the course from which students were recruited, AM and RP have an active research interest in LICs and beliefs around learning within this context. To mitigate against this, the research team was purposefully constructed from the outset to additionally include a variety of clinicians, educators and researchers that were not directly involved with the course.

## Results

**Fig. 2:**
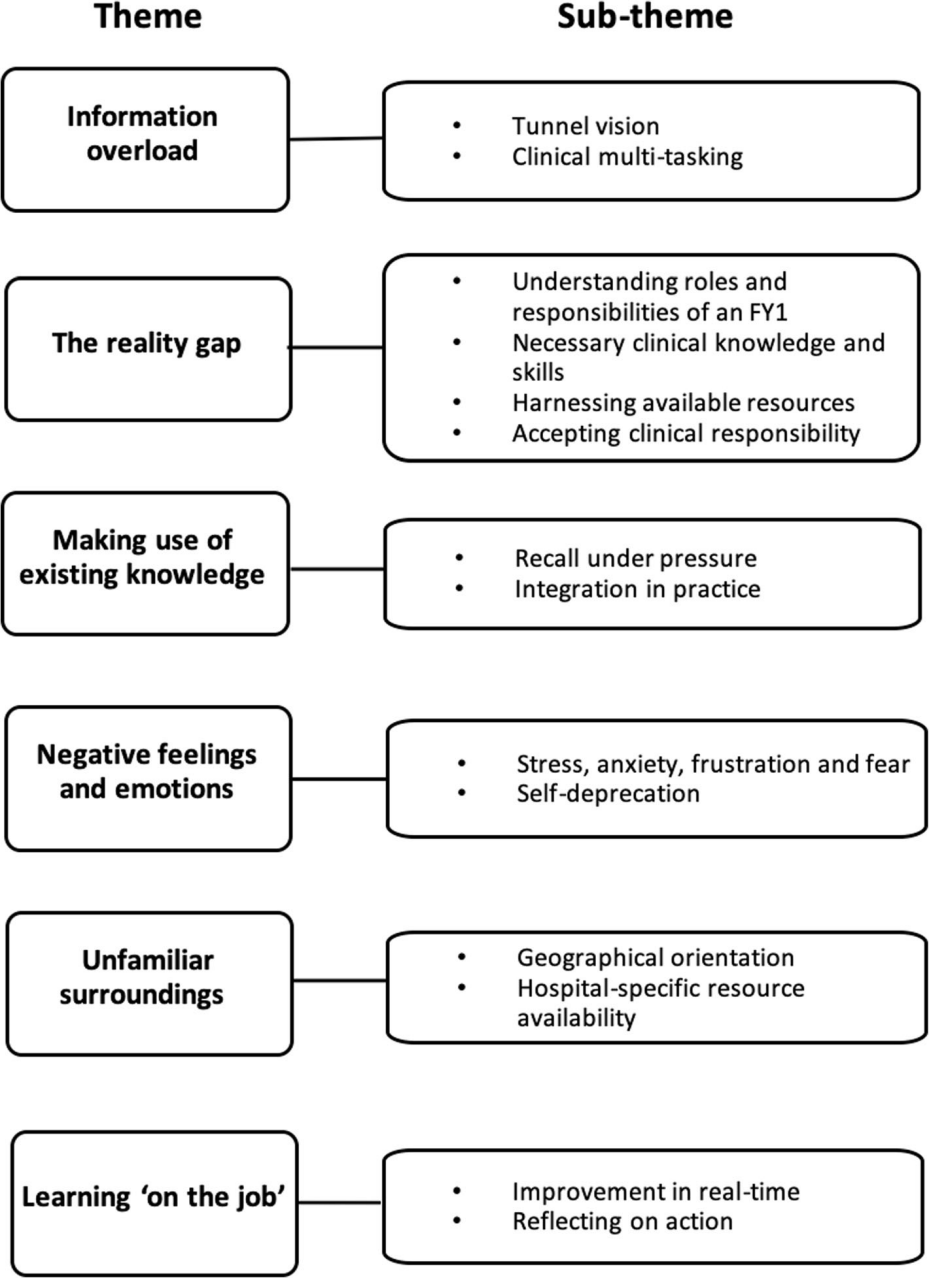
Themes and sub-themes

Findings from the analysis of the data are presented according to six key themes (Fig. 2). Together, these offer insight into the challenges faced by final year medical students during their first experience of working as a newly qualified doctor and aid us in our understanding of the factors affecting undergraduate preparation for clinical practice.

### Information overload

Managing large volumes of information during the on-call simulation was a significant challenge for the students. This was evident both within and between clinical cases. Most notably, when reflecting on individual cases that contained high numbers of interacting information elements, students retrospectively acknowledged a degree of ‘tunnel-vision’ that had impaired their ability to appreciate the bigger clinical picture. For example, faced with a patient who had fallen on the ward, all student pairs focussed in on one particular element of the case – e.g. the fall itself, the new atrial fibrillation (AF) on the electrocardiogram (ECG), or the low blood pressure on examination. Students repeatedly described how it wasn’t until they called their senior for support that they were forced to pause, step back, and consider how the information elements fit together. At this point, they were able to identify the underlying cause of the clinical presentation; this patient’s fall was in fact a result of new-onset fast AF driven by urosepsis.

*‘So, I think we got a bit tunnel vision-ed on the cardio sides of things. And like, didn’t look at the whole picture, because another cause of AF would be urosepsis…If we had been asked, maybe, a question by you, of what we think caused the man’s AF? We’d go, well, there’s this cause, this cause, this group, this group, and think about it logically. But in the moment, when you’ve got seven things going on, we lost that.’ (Student 6)*.

Between cases, students were relatively confident in their ability to prioritise, but struggled to manage multiple patients simultaneously. They described losing track of their patients and the associated clinical tasks as the shift progressed and their attention was pulled in an increasing number of directions.

*‘It was a bit like overwhelming, we were a bit flustered and when we were trying to think about AF and what to do with AF and then someone says can you come to certify a death…You kind of lose track of what you’re thinking.’ (Student 1)*.

*‘Well, we got the death certificate one first and I think, because we were still looking at [the patient], we said, okay well we’ll do it, we’ll go after we’ve just, sort of, sorted everything out… So then, after that, [a bleep about another patient] came through and then we, kind of, forgot about it.’ (Student 12)*.

### The reality gap

Reflecting on their experience of working as an FY1, students repeatedly described how the simulation had enabled them to gain greater insight into the realities of working as a junior doctor through active participation and first-hand experience. A gap became apparent between students’ prior understanding of the job requirements, and their experience of the on-call simulation.

*‘I think doing [final year] so far, I was actually feeling quite good about FY1 because I knew how to write the notes, do a discharge summary, know how to prescribe, but actually, I don’t know if I’m dreading my first on-call now just because I never realised the sheer amount of stuff you have to deal with.’ (Student 10)*.

Students specifically referenced limitations in their knowledge of the roles and responsibilities of an FY1 doctor. During the debrief, they frequently questioned the observers on what an FY1 would be expected to do independently in a given set of circumstances; students were seemingly more focussed on what was considered the ‘right’ or the ‘wrong’ way to behave, over striving to practice within the limitations of their own competencies.

*‘Are we allowed to prescribe blood as FY1’s? …Really?…Okay, I didn’t know that.’ (Student 16)*.

*‘I don’t know if we should’ve asked anyone about stopping the Warfarin?’ (Student 8)*.

*‘I think I was a bit at a loss personally. I was a bit like, I don’t know if I can make that decision… As in terms of whether to stop the Lantus or not. And I was like, should I have enough clinical knowledge to say that?’ (Student 12)*.

Experiencing high levels of uncertainty throughout the on-call shift, newfound deficits in the necessary clinical knowledge and skills were exposed as students stepped into the shoes of the doctor for the first time. These varied between individuals, spanning a wide range of topics and practice areas relevant to a typical on-call shift.

*‘I think we had the ideas though, but we didn’t know how to translate them into practice…what would you actually do or write it down.’ (Student 15)*.

*‘I feel like with some of these things, like when you see people on the team doing it, you think, oh yes, that makes sense. I’d do that, or I know I’m doing that. But until you actually do it you don’t really like pay attention to the little things that are really important, like when do you ask for an ECG?’ (Student 2)*.

Students lacked awareness of the resources available to FY1 doctors faced with such uncertainties in practice, such as specific written guidelines and protocols. Building on this, they reflected on deficits in their understanding of the roles and expertise of surrounding healthcare professionals. This limited the ability of students to work effectively within the multidisciplinary team, impacting delegation practice in particular.

*‘I think as students because we’re so exam-focused, we think we need to have everything memorised. But it’s actually really useful to know that we can use the guidelines.’ (Student 1)*.

*‘And then the death certificate, I wasn’t too sure. So, we got there, and they said someone else had done it. So, obviously someone else could have tried, but I’m not sure who we would pass that off to?’ (Student 8)*.

In taking on a role within which they were no longer ‘just a medical student’, individuals were challenged with managing levels of clinical responsibility higher than those experienced previously. In doing so, the reality of accountability appeared to heighten the sense of uncertainty previously described.

*‘And I think sometimes even when we shadow doctors, there’s always an element of… Unless they’ve said right, you’re taking this patient and I’m going to stand in the corner not do anything, there’s always that element of you can hide behind the doctor. Even if you watch them and you are learning, you’re not doing it. It’s just so different.’ (Student 1)*.

### Making use of existing knowledge

In addition to those areas of practice that students admitted to lacking understanding or awareness of, individuals described difficulty accessing and applying existing knowledge within the on-call environment. Working under high pressure, students struggled to recall previously learned information and frequently referenced feeling forgetful.

*‘In these times, when you’re stressed and there’s a lot going on. I get very forgetful. I guess it’s somewhat normal. But I do find myself thinking something very clearly. And then ten minutes later, I completely forget.’ (Student 17)*.

*‘What’s causing the INR to shoot up? My mind just went blank. I just thought, either too much warfarin or I don’t know.’ (Student 10)*.

Simultaneously managing a variety of medical and surgical patients, students were tasked with retrieving and applying information from a range of different specialties within a single on-call shift. They found it challenging to integrate clinical knowledge in practice that had previously been learnt in silos.

*‘… we went from warfarin to sepsis to hyperkalaemia, upper GI bleed. Then you’re thinking about end of life, certifying a death…it just shows you that you can’t just see cardiology or respiratory, gastro, you actually have to think of everything integrated.’ (Student 9)*.

### Negative feelings and emotions

Student debriefs revealed that very high levels of negative emotion were present during the simulation. These were primarily centred around stress, anxiety, frustration and fear, evoked by taking on the role of a newly qualified doctor. Individuals reflected on how this negatively impacted their performance, often limiting their ability to focus; one student even expressed how the high level of stress led him to consider leaving the simulation altogether mid-way through.

*‘You can’t focus with the stress…’ (Student 13)*.

*‘Everything was going wrong. There was actually a point where I thought, I’m off. I need to get out of here. He can deal with it. I physically cannot take any more. I was so close to going I can’t do this, I’m leaving.‘ (Student 10)*.

Students were highly self-deprecating. Repeatedly criticising their own knowledge, skills and behaviours, they expressed notably low levels of confidence. Students described how this had affected their decision making and actions as a junior doctor on-call, often referencing feelings of embarrassment or inadequacy on escalating to a senior member of the team or delegating a task to another healthcare professional.

*‘But I sort of always feel like… I’m very worried about calling people, being like, ah, I’m just wasting their time. Especially when we called to just double check what we were doing.’ (Student 18)*.

*‘…we did say we were going to [escalate to the medical registrar] and then we were like…Oh, it’s embarrassing.’ (Student 2)*.

### Unfamiliar surroundings

Though students had previously been on placements at the hospital within which the simulation was run, they still eluded to the challenges associated with an unfamiliar environment. Students struggled to orientate themselves on wards they had not previously worked on, describing difficulties in locating patients’ notes, staff members and guidelines.

Building on this, students were not familiar with existing hospital-specific resources. Differing from hospital-to-hospital, these encompassed written protocols, drug charts and multidisciplinary team member availability. This added to students’ workload as they navigated the working environment.

*‘I wasn’t sure about the availability of resources to do that in the evening.’ (Student 6)*.

*‘The thing is you fill out your name and then there’s some tick box and things [on the protocol]. But then when I got really confused was when what do we tick? Do we tick when we’ve done it? Do we tick if we want it done? I know they’re really silly things that I probably should know but it’s just the first time you see…’ (Student 2)*.

### Learning ‘on the job’

Though the aim of our study was not to evaluate the use of simulation as a learning tool, our data suggests students’ learning throughout the simulation and reflective debrief. Both students and observers commented on improvements in student performance as the on-call progressed, and students described how they were able to apply learning from within the simulation itself as the shift continued to evolve.

Importantly, the debrief created an opportunity for students to reflect on their experience of working as a junior doctor for the first time. As previously eluded to, retrospective discussions with their observers enabled students to learn from their actions and decisions made during the simulation.

*‘I think at the beginning I had no idea what to do. I wasn’t enjoying it then. But then I worked out a structure. It’s nice to think through a case…What am I going to do? Have I covered every single thing with the patient?’ (Student 9)*.

## Discussion

**Fig. 3:**
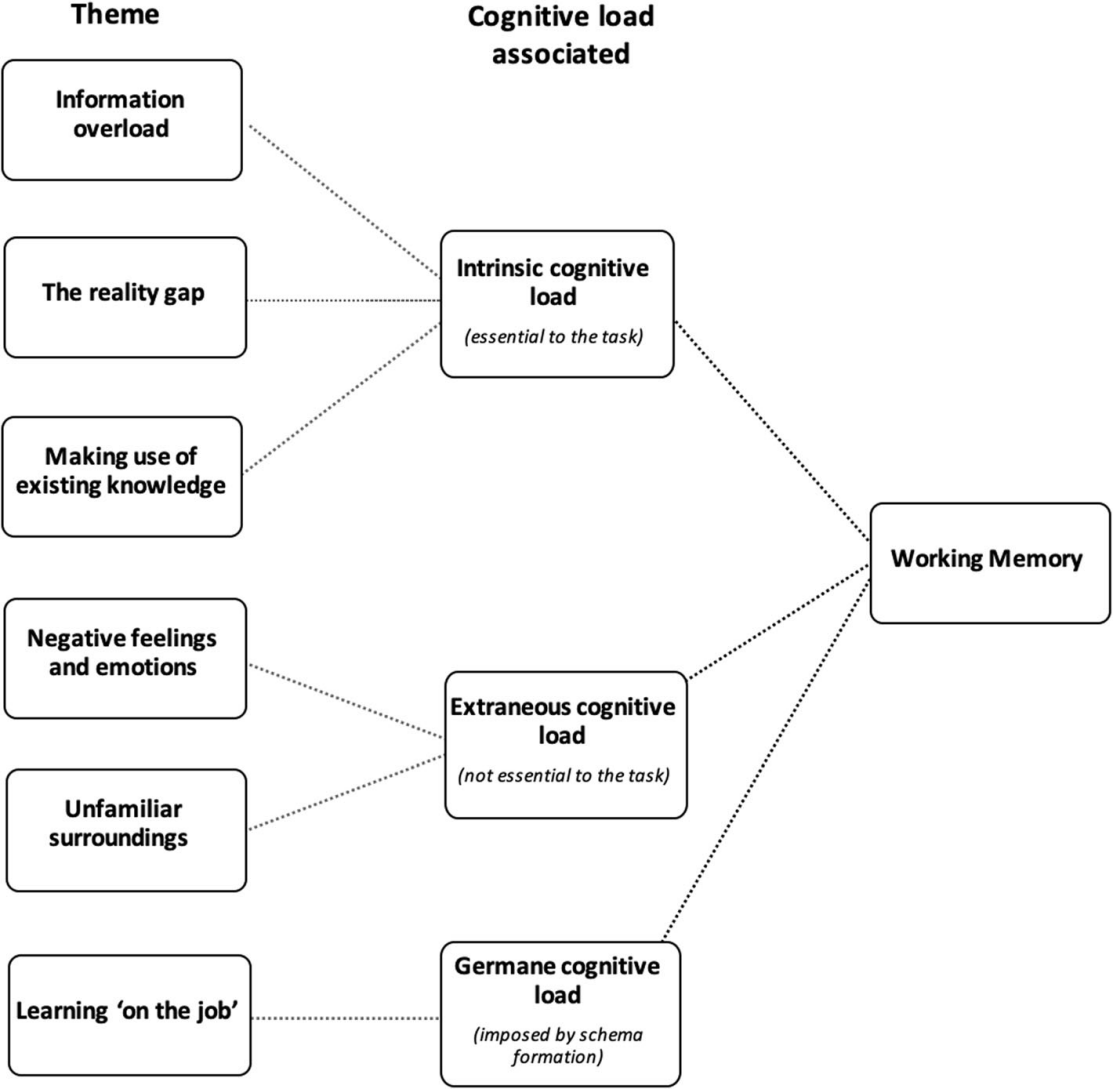
Sources of cognitive load imposed on the learner’s working memory during the on-call simulation. Within the context of CLT, our six key themes (left) can be seen to impose different types of cognitive load (middle) on the working memory of the learner (right) when practising as a newly qualified doctor for the first time

The thematic analysis of transcripts from the simulated on-call debrief allows novel insight into the challenges faced by final year medical students in their preparation for work as newly qualified doctors. In using CLT as a theoretical lens with which to conceptualise the data (Fig. 3), we can begin to understand how cognitive load may be optimised within this context and, in doing so, identify adaptations to undergraduate curricula that may be necessary to better support students’ learning throughout medical school in their preparation for clinical practice.

Traditionally, optimising cognitive load focuses on reducing extraneous load so that the individual may allocate working memory resources to the management of intrinsic cognitive load, and to engage in learning [13]. In keeping with this, we had originally anticipated that students completing the simulation would primarily be challenged by the high burden of extraneous cognitive load associated with an on-call shift, such as bleeps, interruptions and surrounding distractions. We were therefore surprised to find that the overwhelming focus of the dataset was in fact on factors seen as contributing to intrinsic cognitive load, with an emphasis on high numbers of interacting information elements and students’ knowledge availability. Furthermore, of the findings consistent with extraneous cognitive load, it was in fact a strong sense of negativity and self-deprecation that dominated the data. In light of these findings, we subsequently expand on three key discussion points.

### Enhancing the expertise of the learner

**Fig. 4:**
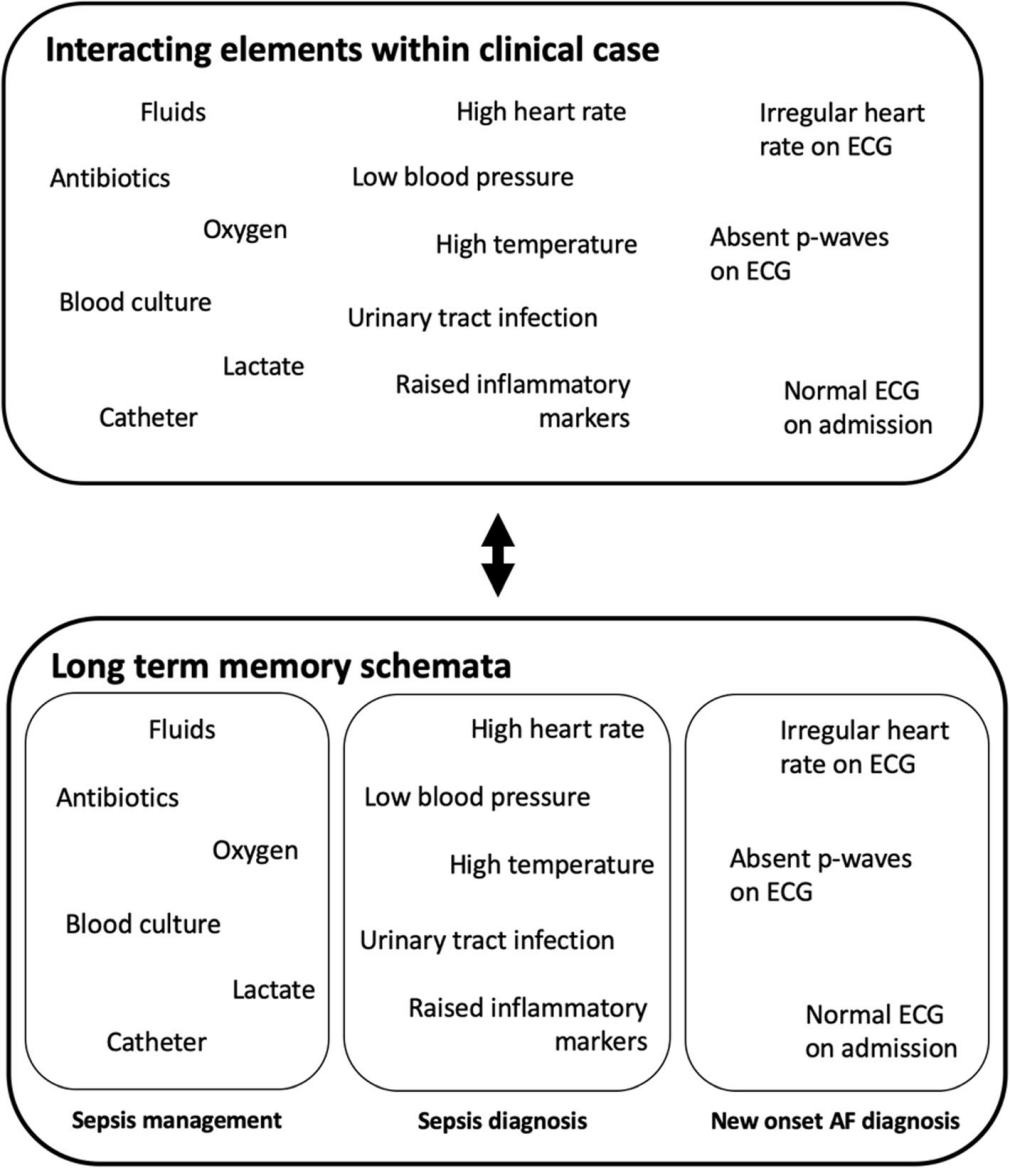
Schemata utilised in the assessment and management of patient presenting with urosepsis by an experienced learner. Having previously developed schemata in long term memory (bottom) relative to elements central to the task (top), intrinsic cognitive load imposed on the experienced learner is reduced as information can be retrieved and processed as fewer single elements in working memory

Young et al. [12] describe how the intrinsic load is dictated by the following factors: number of information elements, degree of element interactivity, and proficiency of the individual learner. In practice, the number and interactivity of information elements within an on-call shift are inherent to the task and cannot be controlled; they are determined by individual patient issues, clinical presentations and context. The role of faculty in preparing students therefore lies in minimising cognitive load imposed by these factors by enhancing the ‘expertise’ of the learner in advance; i.e. increasing the availability and automaticity of the relevant cognitive schemata in long term memory. Schemata are subsequently activated in the working memory as one single element, as opposed to multiple interacting elements, thus reducing intrinsic cognitive load [12]. Figure 4 illustrates this concept within the context of the on-call simulation, using the urosepsis case previously described in the results.

Building on this, Young et al. [12] go on to describe the effects of fragmentation of an educational programme (i.e. de-contextualising real-life tasks by splitting them into sections that are taught at different stages in the curriculum – often seen within undergraduate medicine) on schemata formation. Consistent with our data, in transferring learning from this approach into clinical practice, individuals struggle to retrieve and organise all of the separate schemata stored in long term memory necessary to complete the task. Young et al. [12] therefore outline the benefits of ‘whole-task’ training approaches on the regulation of working memory processes, promoting ‘complex learning’ of the necessary clinical knowledge, skills and behaviour in an integrated way that is representative of real life.

Authentic learning experiences, appropriate to the level of the learner, are therefore key in addressing the challenging transition from medical student to junior doctor. This aligns with recommendations from a seminal multicentre study, previously commissioned by the GMC to explore and address concerns around preparation for practice, endorsing the use of experiential learning within undergraduate medical curricula [4]. Other examples of medical education initiatives employing a ‘whole-task’ training approach include entrustable professional activities [14], LICs [15] and the transformation of early clinical experiences through intensifying student responsibility within limited and controlled domains [16]. Further study into the longer-lasting educational impact of these initiatives, compared to more traditional teaching interventions aimed at the undergraduate-postgraduate transition, would be of great value.

### Navigating negative emotions

Existing literature describes the complex relationship between emotions and learning; learning and performance are thought to be impaired under conditions of heightened negative emotions through the generation of extraneous cognitive load [17]. We identified high levels of stress and anxiety, as well as a strong sense of self-deprecation, amongst the students as they took on the role of a newly qualified doctor for the first time.

Evoked by challenges intrinsic to an authentic on-call shift, and arguably unavoidable, medical school curricula must therefore support students in learning to manage these feelings, strengthen their resilience within the workplace, and develop confidence in their ability to recognise and practice within their own limitations. Incorporating reflective practice into undergraduate clinical experiences from an early stage promotes identification of these challenges by both students and faculty so that they may be acknowledged and addressed throughout medical school.

Of note, extraneous load associated with a new working environment, as is frequently encountered as a junior doctor given the frequent rotation of job posts, is likely to be addressed more successfully at site-specific inductions as opposed to within the undergraduate curricula.

### Supporting lifelong ‘learning while working’

In minimising intrinsic and extraneous load, not only is there a reduction in the risk of overloading the working memory capacity of the learner, but an increase in the resources available for germane load associated with learning itself [12]. This involves the arrangement of new information elements with schemata that already exist in long-term memory; evidenced within our data, students consistently reflected on their learning from the experience and the resultant improvement in performance as the simulation progressed. This is highly significant for both medical students and junior doctors as, in essence, a career in medicine demands lifelong learning at work [18].

Amongst other suggestions on how best to develop suitable learning for use throughout working life as a doctor, Teunissen et al. highlight the value of capitalising on ‘on the job’ learning opportunities within the workplace (i.e. learning while working) [18]. In this regard, by striving to reduce the intrinsic and extraneous cognitive load of new graduates using the strategies described above, germane resources may be increased. This holds potential to enhance individuals’ ‘learning while working’ and therefore support their continued professional development as FY1s and beyond.

### Limitations

We recognise that this is a small-scale study involving LIC students from a single UK medical school, therefore restricting generalisability of the data. A larger-scale study spanning more than one medical school would account for increased variability in student demographics and personal experience – both factors that are likely to influence students’ preparedness for clinical practice. We also recognise that the intensity of resources in developing and running an on-call simulation of this scale may limit reproduction of the work. Finally, we acknowledge a degree of additional extraneous cognitive load imposed on students secondary to inauthentic features of the simulation; these include the use of a manikin, students working in pairs, and an observer following students at all times.

## Conclusions

The combination of high fidelity on-call simulation, close observation and personalised debrief is unique. It offers a novel insight into the difficulties faced by undergraduates in their preparation for work as a junior doctor, and a valuable opportunity to encourage reflection on challenges that may otherwise be missed or remain unrecognised by students. To the best of our knowledge, this is the first study investigating preparedness for practice in final year medical students using qualitative data from reflective debrief after a simulation of this nature.

Focussing our investigation around an authentic on-call shift, representative of those encountered by newly qualified doctors, we have used the CLT as a theoretical lens to better understand how learning and preparation may be optimised within this context. In particular, our findings endorse ‘whole-task’ training approaches within undergraduate medical curricula, to promote integrated learning and encourage the development of schemata in long-term memory. Furthermore, this work highlights the importance of supporting students in developing resilience, managing negative emotion and recognising their own limitations in practice. Alongside robust site-specific inductions, together these recommendations promote optimisation of cognitive load in students’ preparation for clinical work, better supporting them in the challenging transition from medical student to junior doctor and beyond.

## Data Availability

The datasets (interview transcripts) generated and analysed during the current study are not publicly available. This is in accordance with the written informed consent of student participants and the ethical approval granted for the study. The authors would be happy to provide samples of the dataset to the reviewers, should this be required.

## Abbreviations

AF: Atrial Fibrillation
CLT: Cognitive Load Theory
ECG: Electrocardiogram
FY1: Foundation Year 1
GMC: General Medical Council
ICSM: Imperial College School of Medicine
LIC: Longitudinal Integrated Clerkship

## Authors’ information

NH is an Honorary GP Teaching Fellow within the Department of Primary Care and Public Health at Imperial College, London. Currently in her final year of GP training, she is developing an academic interest within undergraduate medical education.

H-CY is an internal medicine trainee at Imperial College Healthcare NHS Trust. She recently completed an academic foundation training post in the Department of Primary Care and Public Health at Imperial College, London.

MF is a Senior Teaching Fellow in Education Research at Imperial College, with interests in health professions education, diversity in higher education and public health.

RP is a GP and Deputy Director of the Medical Education Innovation & Research Centre at Imperial College, with an interest in longitudinal integrated clerkships and medical student preparedness for clinical practice.

AM is a GP and Deputy Director of Medical Education at the University of Buckingham, focussing on the development of their Crewe Campus. His interests lie in educational authenticity, longitudinal integrated clerkships and interprofessional learning.
